# Hepatic inflammatory myofibroblastic tumor: One case report

**DOI:** 10.3389/fsurg.2022.902753

**Published:** 2022-08-04

**Authors:** Lei Shen, Zixuan Yang, Ruibo Ding, Wei Wei, Yechuan Xu

**Affiliations:** Department of Hepatobiliary and Pancreatic Surgery, The First Affiliated Hospital of Anhui Medical University, Heifei, China

**Keywords:** inflammatory myofibroblastic tumor, hepatic, case report, review, cancer

## Abstract

**Introduction:**

Hepatic inflammatory myofibroblastic tumor (HIMT) is a junctional neoplastic lesion of mesenchymal tissue origin that can sometimes become locally invasive and even metastasize or recur. Therefore, the diagnosis and treatment of HIMT is particularly important. However, hepatic inflammatory myofibroblastic tumor lacks a specific clinical presentation and typical imaging manifestations, thus posing a difficulty for us to diagnose and treat this disease.

**Case Presentation:**

We report here a very rare surgical case of hepatic inflammatory myofibroblastic tumor (HIMT) in a 41-year-old female who was admitted to the hospital for more than half a month for a liver-occupying lesion with fever found on physical examination.After discussion with the hepatobiliary and pancreatic surgery team, we decided to perform surgical treatment. The final postoperative pathology confirmed hepatitis myofibroblastoma.

**Conclusion:**

Our review of the domestic and international literature revealed no significant progress in the diagnosis and treatment of this disease, so we report here a case of surgical treatment. One of our aims in this case report is to highlight the efficacy of surgical treatment in HIMT. HIMT is extremely rare and difficult to diagnose. Due to their intermediate biological behavior, surgical resection should be performed whenever feasible and patients should be followed-up in order to detect recurrence and metastasis as early as possible.

## Introduction

Hepatic inflammatory myofibroblastic tumor (HIMT) is a very rare tumor in clinical practice.Its etiology and pathogenesis are not clear at present, and its clinical manifestations and imaging data are non-specific, so it poses a difficulty in clinical diagnosis and treatment. This rare tumor was first reported in the lung, but also can occur in a variety of tissues and organs in the body (1). HIMT was initially thought to be an inflammatory tumor, but was later found to have the ability to recur and metastasize, so it is now considered to be a true tumor (2, 3).

Because the disease is rare and lacks specific clinical manifestations and imaging features, it is easily misdiagnosed as hepatocellular carcinoma when patients have risk factors for hepatocellular carcinoma. Therefore, how to improve the accuracy of the diagnosis and find the best treatment is the question we have to think about. However, given the characteristics of the disease, it is almost impossible to obtain a definitive diagnosis without pathological examination. When hepatitis myofibroblastoma is suspected, we recommend a puncture biopsy or surgical resection to clarify the diagnosis. Currently, there is a great debate on the treatment of hepatitis myofibroblastoma, with some advocating non-surgical treatment and others suggesting surgical treatment (4). Thus, this poses a difficult problem for clinicians.

We have reviewed several Chinese and English literature on hepatic inflammatory myofibroblastic tumor in recent years. and since this case is rare and not much literature is involved, here we briefly report a surgical case of this disease. In this case report, we have two objectives: the first to highlight that HIMT should be considered by clinicopathology in the diagnosis of liver masses. The second is to highlight the effectiveness of surgical treatment in hepatic inflammatory myofibroblastic tumor.

## Case report

**Figure 1:**
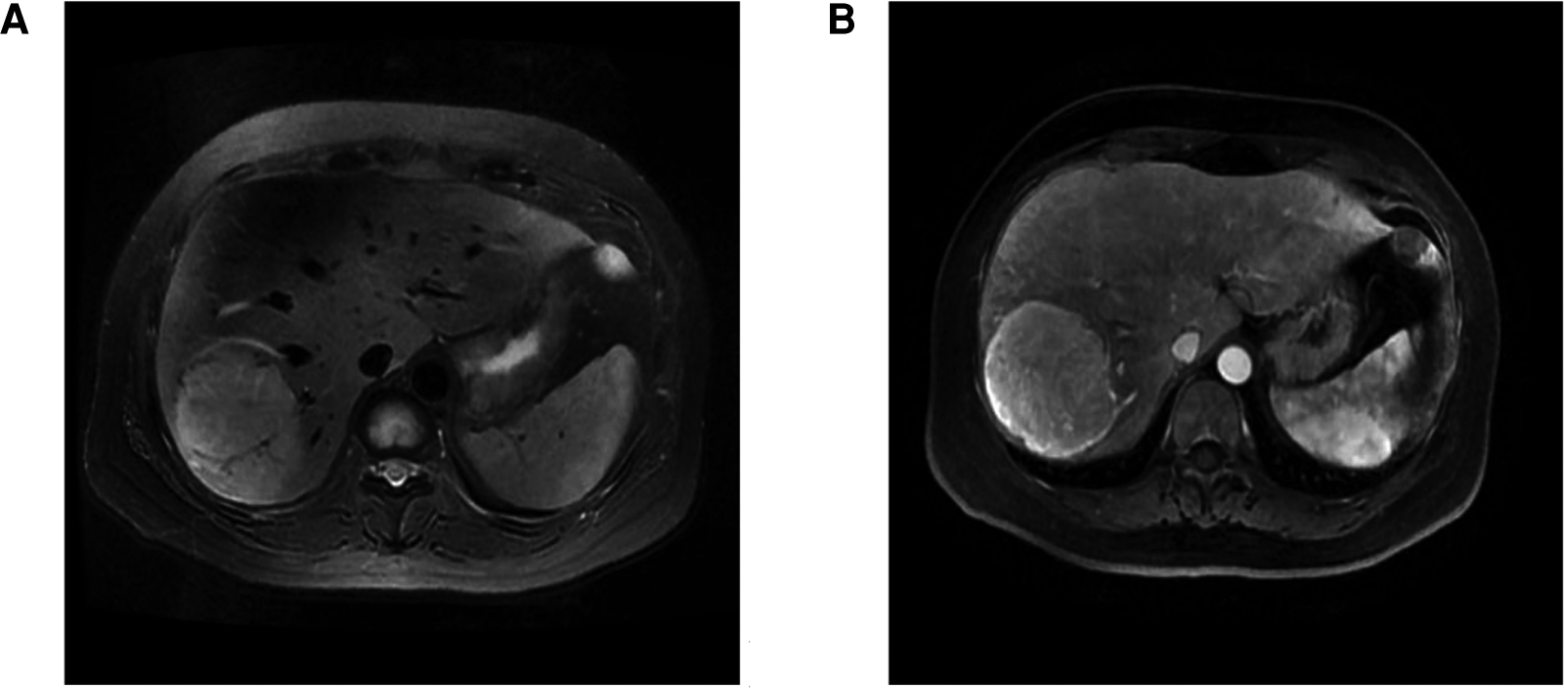
MRI of inflammatory myofibroblastoma of the liver. (**A**) shows the MRI plain T2-weighted image (T2WI). (**B**) shows the enhanced scan image.

**Figure 2:**
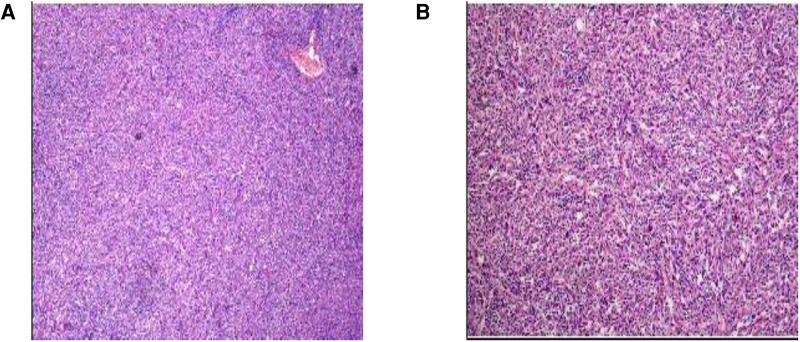
Pathological picture of inflammatory myofibroblasts in the liver. A large number of spindle cell proliferation in the liver tissue is seen in the figure.

The patient, a 41-year-old female, was admitted to the hospital with fever for more than half a month after a physical examination revealed an occupying liver lesion. The patient's physical examination revealed liver occupancy but no special treatment was done. Subsequently, she was seen in the hospital because of a review that revealed a larger swelling and a fever that developed half a month ago. The patient's past history was unremarkable, with no history of hepatitis B or C, and there, were no significant positive signs on abdominal physical examination. After admission to the hospital to complete blood and imaging tests, our blood test results showed: White Blood Cells(WBC):11.02*10^9^/L, Alpha fetal Protein (AFP):0.820 ng/ml, Carcinoembryonic antigen (CEA):0.91 ng/ml, Carbohydrate antigen125 (CA125): 22.19 U/ml, Carbohydrate antigen199CA199:1.69 U/ml, Calcitonin original (PCT): 0.12 ng/ml, C-reactive protein(CRP): 182.12 mg/L. The patient's liver function was basically normal, no bacterial growth was found in the blood bacterial culture and no fungus was found in the fecal and urine microscopy. Our enhancement MRI showed an abnormal signal in the right lobe of the liver, considering spindle cell tumor or tumor of mesenchymal origin (Figure 1). We evaluated and staged the tumor by chest CT and abdominal MRI, and no signs of distant metastasis were seen. Combining with the patient's clinical manifestations, physical examination and ancillary examinations, the hepatic malignant tumor could not be distinguished from other benign tumors of the liver. Finally, after team discussion, we proceeded to perform right hemicolectomy. The postoperative pathology of this patient was diagnosed as inflammatory myofibroblastoma and the immunohistochemistry was: ALK-1(−), CD23(−), CD21(−), CD30(−), CD34 (endothelial+), EMA(scattered+), SMA(−), Vim(+), Ki-67(15%) (Figure 2). The patient was finally diagnosed hepatic inflammatory myofibroblastic tumor.

The patient was discharged in good general condition, the laboratory parameters were normal and we recommended regular follow-up in the outpatient clinic.

The last outpatient follow-up was in April 2022 (Table 1), and the patient had normal tumor markers. Ultrasound and MRI showed no signs of recurrence. During the follow-up period, the patient was compliant and was able to attend regular outpatient appointments without adverse events.

**Table 1:** Timeline.

August 01, 2020	First symptoms: abdominal pain with fever
August 15, 2020	CT abdomen: right lobe of liver is occupied, consider hepatocellular carcinoma
August 18, 2020	Hospitalization
August 22, 2020	MRI of abdomen: abnormal signal in the right lobe of liver, considering spindle cell tumor, size about 9*9*6 cm
August 31, 2020	After discussion with the hepatobiliary and pancreatic surgery team, surgical treatment was performed
September 01, 2020	Surgical treatment
September 11, 2020	Discharge from hospital
April 04, 2022	Latest follow-up

## Discussion

Hepatic inflammatory myofibroblastic tumor was once first found in lung tissue, but as the level of ancillary testing has improved, it has been reported in salivary glands, stomach, spleen, skin, and other tissues and organs. Inflammatory myofibroblastoma located in liver tissue is less common and is a rare junctional tumor of mesenchymal origin called hepatitis myofibroblastoma. It has been reported in the literature that hepatitis inflammatory myofibroblastic tumors can occur at any age, mostly in children or young adults, but no significant differences in gender have been observed (5). The pathogenesis of the disease is still unclear, and some reports in the literature suggest that its occurrence is related factors such as infection, trauma, hormone therapy, and these factors stimulate the proliferation of myofibroblasts in the liver (6). In addition, the development of HIMT is also associated with a variety of inflammatory diseases throughout the body, including chronic cholangitis, autoimmune diseases, and gout (7). The patient in this case was a young female, she denied history of trauma, history of hepatitis and schistosomiasis, and was febrile as the main symptom. Although the main symptoms of inflammatory myofibroblastic tumor are pain in the right upper abdomen, fever, and jaundice, they are not specific clinical manifestations, making it difficult to accurately diagnose the disease. Similarly, hepatitis myofibroblastoma lacks specificity on imaging and usually appears as a hypoechoic mass on hepatobiliary and pancreatic ultrasound, rarely showing hyperechogenicity. It has been reported in the literature that the majority of HIMT patients exhibit a malignant tumor enhancing pattern on ultrasonography, therefore HIMT cannot be completely excluded when this pattern is demonstrated on ultrasonography. The disease mostly appears as a hypointense shadow on CT, with a variety of enhancement patterns, including total tumor filling, marginal enhancement, segregated enhancement, and no enhancement. It has been shown that the thickness of the tumor envelope is significantly thicker than that of hepatocellular carcinoma on CT images of patients with hepatitis myofibroblastoma. Therefore, the periportal retraction sign, which is specific to hepatic malignancies, has also been used as one of the means to identify malignant tumors (8). The disease should be differentiated not only from hepatic malignancies but also from liver abscesses, hepatic lymphomas, isolated necrotic nodules, and liver metastases. MRI is similar to CT in terms of the mode and degree of enhancement. T1W1 is often a low signal and T2W2 is often a medium to high signal in MRI.

Because hepatitis inflammatory myofibroblastic tumor is rare and lacks specific clinical manifestations and imaging features, it is easily misdiagnosed as hepatocellular carcinoma when patients have risk factors for hepatocellular carcinoma. From this case, we can see that the patient was admitted with fever for half a month and treated with various antibiotics before surgical treatment, but the effect did not improve significantly. Moreover, the patient's tumor markers were normal, but magnetic resonance showed an occupancy in the right lobe of the liver, so it was difficult to distinguish it from liver malignancy. Finally, after preoperative discussion and the patient's strong desire for surgery, we performed a right hemihepatectomy and the diagnosis was confirmed by postoperative pathological examination. However, none of the above imaging tests are specific, which makes the diagnosis of hepatitis myofibroblastoma difficult.

Overall, hepatitis inflammatory myofibroblastic tumor is a junctional neoplastic lesion, and there is controversy over the treatment of this disease, which includes surgical treatment, high-dose steroid hormones, nonsteroidal anti-inflammatory drugs, and conservative treatment. It has been argued that partial hepatectomy should be the best treatment (9). However, a multicenter study also showed that 27 patients with hepatitis myofibroblastoma had tumor shrinkage after conservative treatment (10). In recent years, a case of a patient with hepatic occupancy was identified, and because no evidence of malignancy was found by preoperative liver puncture, close observation was chosen for follow-up, and the tumor was eventually found to have completely disappeared (11). After combining the above treatments, we opted for surgery considering that the patient's febrile symptoms did not improve due to the progressive enlargement of the tumor and the use of multiple antibiotics. The patient recovered well after surgical treatment, and the patient's temperature returned to normal the day after surgery and did not return after antibiotic downgrade use. From the diagnosis and treatment of this case, we have a deeper understanding of the diagnosis and treatment of hepatitis myofibroblastoma, although there are some case reports in which the tumor was found to regress after oral administration of non-steroidal anti-inflammatory drugs or antibiotics. However, if patients experience worsening of symptoms, tumor enlargement, or even compression of surrounding tissues, prompt surgical treatment should be performed. Although there are reports of recurrence of HIMT after surgery, the majority of patients currently have a good prognosis after surgical treatment, and there are very few cases of recurrence after surgery, so the author believes that surgery is still the best option for the treatment of this disease.

## Patient's statement

We contacted the patient in October 2020 and asked her for her views and insights on our treatment. The patient said she was very satisfied with the treatment her doctors had chosen for her throughout the treatment process. Before the operation, she had been suffering from abdominal pain and fever. This caused her distress and seriously affected her daily work life. When asked how her life has been since the surgery, she said she has returned to work and is making regular visits to the hospital. She said the surgery saved her life and wanted to stress how grateful she was that her condition was treated successfully. And she said she was pleased her case would be submitted for publication and believed it would help find treatments for patients with the same diagnosis.

## Data Availability

The original contributions presented in the study are included in the article/Supplementary Material, further inquiries can be directed to the corresponding author/s.
